# BOULE, a Deleted in Azoospermia Homolog, Is Recruited to Stress Granules in the Mouse Male Germ Cells

**DOI:** 10.1371/journal.pone.0163015

**Published:** 2016-09-15

**Authors:** Byunghyuk Kim, Kunsoo Rhee

**Affiliations:** Department of Biological Sciences, Seoul National University, Seoul, Korea; University of Macau, MACAO

## Abstract

High temperature adversely affects normal development of male germ cells in mammals. Acute heat stress induces the formation of stress granules (SGs) in a set of male germ cells, and the SGs have been proposed to protect those cells from heat-induced apoptosis. DAZL, one of DAZ (Deleted in Azoospermia) family proteins, was shown to be an essential component of SGs, which is required for SG formation in the mouse testis. In the present study, we asked whether BOULE, the founding member of DAZ family proteins, is a component of the SGs. We show that BOULE is recruited to the SGs upon heat stress, and that these SGs are developmental stage-specific. These results suggest that DAZ family proteins may have conserved roles in the SGs of male germ cells.

## Introduction

*DAZ* was originally identified as a frequently deleted gene on the Y chromosome of infertile men [1]. Later, it was shown that the canonical Y chromosome contains four copies of *DAZ* genes, which are highly polymorphic among individuals [2–5]. In addition, two autosomal *DAZ* homologs, *BOULE* and *DAZL*, have been found in human, together comprising *DAZ* gene family [6,7]. Due to its conservation across the metazoan lineages, *BOULE* is considered a founding member of the *DAZ* gene family [7–9].

Studies on expression of the *DAZ* family genes have identified predominant expression in germ cells. *DAZ* is expressed exclusively in male germ cells of human and old world primates [10,11]; *DAZL* is expressed in the germ cells of both sexes in mice [12]; *BOULE* is detected preferentially in male germ cells across various taxa [9]. Consistent with these results, depletion of the *DAZ* family genes leads to defects in germ cell development of many organisms, including worms (*Caenorhabditis elegans*) [13], fruit flies (*Drosophila melanogaster*) [14], sawflies (*Athalia rosae*) [15], frogs (*Xenopus laevis*) [16] and mice (*Mus musculus*) [12,17]. Using human embryonic stem cells, it was shown that *DAZ* family genes function in germ cell formation and meiotic progression [18]. Therefore, all *DAZ* family genes are regarded as critical for germ cell development.

Mammalian testes should be maintained 2–7°C below core body temperature for a normal process of spermatogenesis. Severe or repetitive heat exposures often induce male subfertility or infertility due to reduced sperm output and qualities [19]. In a heat stress condition, mammalian male germ cells show a variety of changes in cellular events including stress granule (SG) formation, DNA damage and apoptosis (Reviewed in [20]). SGs are cytoplasmic aggregates composed of messenger ribonucleoprotein (mRNP) complexes, and their formation is triggered by various stresses such as heat, hypoxia or oxidative stress [21,22]. Functionally, SGs are considered a regulatory site for mRNP remodeling and translation and also enhance cell survival by sequestering pro-apoptotic signaling proteins during stress conditions [23–26]. We have previously found that an RNA-binding protein DAZL is a SG component, regulates its formation and thus protects a set of mouse male germ cells from heat-induced apoptosis [27]. This raises a question of whether DAZ family proteins, as conserved germ cell-specific translational regulators, share these properties.

The mouse genome includes two *DAZ* family genes, *Dazl* and *Boule*. Here, we examine whether BOULE is a SG component like DAZL in mice. We show that BOULE is relocated to SGs in a heat stress condition in a developmental stage-specific manner. We propose that all DAZ family proteins may function similarly in SGs.

## Materials and Methods

### Animals and heat treatment

Mice were housed and treated under approval of Institutional Animal Care and Use Committee at Seoul National University (SNU-090320-3). FVB adult male mice (8~10 weeks) were anesthetized with tribromoethanol (Avertin, Sigma-Aldrich) at a dose of 250 mg/Kg. The lower third of the body was placed in a water bath at 42°C for 20 min (heat stress, n = 3) or mock-treated (control, n = 3). During the heat exposure, we did not monitor the core body temperature of the mice, but there were no signs of adverse effects such as illness or mortality. After treatment, mice were immediately sacrificed by cervical dislocation under anesthesia, and the testes were isolated for further analyses. All efforts were made to minimize suffering.

To obtain mouse testes without germ cells, we used C57BL/6 inbred *Dazl* knockout mice (*Dazl*^*Tm1Hgu/Tm1Hgu*^) and genotypes were screened by PCR using DNAs isolated from tail tips as described previously [12].

### Plasmids and antibody generation

The HA-tagged human cDNA clones for three DAZ family proteins (DAZ2, DAZL, BOULE) were previously described [4]. A rabbit polyclonal antibody was raised against bacterially expressed GST-BOULE fusion proteins and further affinity-purified using an immunoblot method [28].

### Cell culture and transfection

Cell culture and transfection were performed as described previously [4]. Briefly, 293T cells were cultured in DMEM containing 10% fetal bovine serum, and maintained in 5% CO_2_ at 37°C. Plasmids were transfected using polyethylenimine (Sigma-Aldrich) and the cells were lysed 24 hours after transfection for immunoblot analysis.

### Immunoblot analysis

Decapsulated mouse testes or cultured cells were lysed in 1×SDS sample buffer (50mM Tris-HCl, pH 6.8, 100mM dithiothreitol, 2% SDS, 0.1% bromophenol blue, 10% glycerol). About 20~50 μg of protein were used per lane in a SDS-PAGE gel and transferred into nitrocellulose membranes. The membranes were blocked with 5% skim milk in TBST (20 mM Tris, 150 mM NaCl, 0.1% Tween 20) for 30 min, and then incubated overnight at 4°C with the following antibodies: anti-HA (1:10,000, Sigma), anti-β-tubulin (1:1,000, Sigma), affinity-purified anti-BOULE (1:100) and preimmune serum (1:1,000). After washing three times with TBST, the membranes were incubated with peroxidase-conjugated secondary antibodies for 30 min. After further washing three times with TBST, the peroxidase activity was detected using ECL reagent.

### Immunostaining and Microscopy

Heat- or mock-treated mouse testes were fixed in Bouin’s solution (Sigma-Aldrich) and paraffin-embedded. The tissue sections (5 μm) were deparaffinized and hydrated. Antigen retrieval was performed by microwave boiling for 15 min in 10 mM Tris-HCl (pH 9.0). The testicular sections were then blocked with 3% BSA (bovine serum albumin) in PBST (phosphate-buffered saline with 0.1% Tween 20) for 30 min and incubated for 1 h with the following antibodies: affinity-purified anti-BOULE (1:10), affinity-purified anti-DAZL (1:10) [29], anti-TIA-1 (1:25, Santa Cruz) and preimmune serum (1:1,00). For DAB staining, the sections were incubated with a biotinylated antibody for 30 min, followed by incubation with avidin-biotinylated peroxidase complex (Vector). Otherwise, fluorophore-conjugated secondary antibodies (Invitrogen) were used to detect signals. Counterstaing was performed using hematoxylin or DAPI (Sigma-Aldrich). The slides were observed with a light or fluorescence microscope (Olympus IX51). Images were prepared using a camera (Qicam fast 1394, Qimaging) and ImagePro 5.0 software (Media Cybernetics, Inc.).

## Results

**Fig 1:**
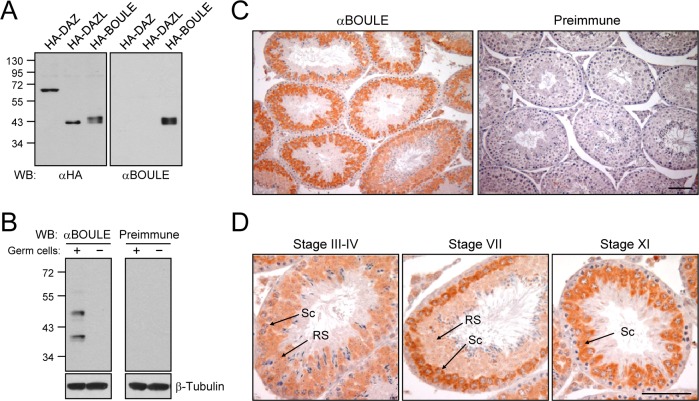
Expression of the BOULE protein in the mouse testis. (A) Specificity of the BOULE antibody. The 293T cells expressing HA-DAZ, HA-DAZL, and HA-BOULE were subjected to immunoblot analyses with the HA and BOULE antibodies. (B) Male germ cell-specific expression of BOULE. Mouse testicular lysates with (wild type) or without (*Dazl* knockout) male germ cells were subjected to immunoblot analyses with the BOULE antibody and preimmune serum. β-Tubulin is shown as a loading control. (C) Immunohistochemical analysis of the mouse testis was performed with the BOULE antibody and preimmune serum. (D) Developmental stage-specific expression of BOULE. The stages of the seminiferous epithelial cycle were determined according to Russell *et al*. (1990) [30]. Sc, spermatocyte; RS, round spermatid. Scale bars, 100 μm.

In the beginning of our study, we sought to detect the BOULE protein in the mouse testis. A polyclonal antibody against a human BOULE fusion protein was raised and affinity-purified. Specificity of the BOULE antibody was examined with 293T cells which expressed the ectopic DAZ family proteins. The results showed that the BOULE antibody specifically recognized HA-BOULE, but not HA-DAZ nor HA-DAZL (Fig 1A). We performed immunoblot analysis with testis lysates of wild type and *Dazl*-deleted mice. The *Dazl*-deleted mouse lacks germ cells in the adult testis [12]. The results showed that the BOULE antibody detected two bands only in the wild type mouse testis (Fig 1B). The lower band of ~40 kDa in size corresponds to the predicted endogenous BOULE protein, while the upper band may be an isoform or posttranslatinally modified version of the BOULE protein [17].

We performed immunohistochemical analysis of BOULE in the mouse testis sections. The BOULE antibody strongly immunostained the cytoplasm of male germ cells at early developmental stages in all the seminiferous tubules (Fig 1C). We carefully observed developmental stage-specific expression of BOULE, based on the staging criteria of the mouse seminiferous tubules [30]. The results showed that BOULE was expressed in spermatocytes at pachytene, diplotene and meiotic stages as well as in round spermatids, with higher expression levels in late pachytene spermatocytes before meiotic division (Fig 1D). Our observation is largely consistent with the previous report [17].

**Fig 2:**
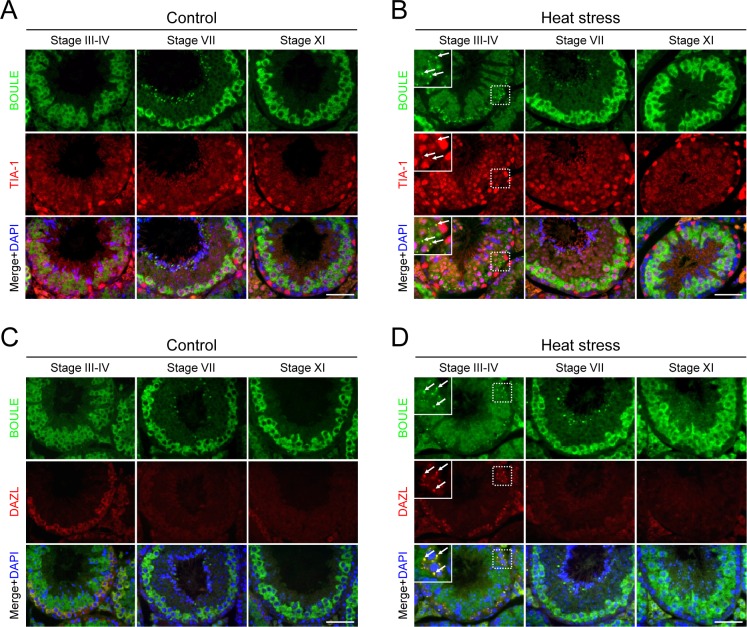
BOULE is recruited to SGs in the heat-stressed testes. Adult testes from control (A and C) or heat-treated (42°C for 20 min) mice (B and D) were immunostained for BOULE (green), along with the SG markers, TIA-1 and DAZL (red). DNA was stained with DAPI (blue). Seminiferous tubules at specific developmental stages were representatively shown. The insets are magnified views. SGs are marked with arrows. Scale bars, 50 μm.

We previously reported that heat stress induces SG formation in a set of male germ cells, and DAZL is localized at the SGs [27]. In order to determine whether BOULE is also a component of SG or not, we immunostained the testis sections obtained from a heat-stressed mouse with the BOULE antibody. In the untreated testis, TIA-1, a marker for SG [31], was localized in the nuclei of somatic Sertoli cells as well as spermatocytes and round spermatids (Fig 2A). Upon heat stress, however, a fraction of TIA-1 signal was relocated to the cytoplasmic SGs of spermatocytes at tubular stages III-VI [27] (Fig 2B). In this condition, BOULE was localized to the TIA-1-positive SGs at the same germ cells (Fig 2B). We observed SG localization of BOULE in all heat-stressed animals (n = 3), but not in untreated animals (n = 3). This result suggests that BOULE is a SG component in heat-stressed male germ cells.

DAZL is a germ cell-specific SG component, and the germ cells expressing DAZL are known to correlate with those containing SGs [27]. We coimmunostained both DAZL and BOULE to compare their expression and SG localization. DAZL is predominantly expressed in spermatogonia and early stages of spermatocytes at leptotene, zygotene and pachytene [12,27], whereas BOULE is expressed in spermatocytes at pachytene, diplotene and meiosis and round spermatids as described above (Fig 2C). The expression of BOULE overlaps with that of DAZL in early pachytene spermatocytes at stages III-VI (Fig 2C). In the heat-stress condition, DAZL was recruited to SGs in the cells expressing DAZL, while BOULE was detected in the SGs of early pachytene spermatocytes (stages III-VI) even though the expression levels of BOULE were relatively weaker in those cells (Fig 2D). This result confirms the SG localization of BOULE, and suggests that SG formation is developmental stage-specific regardless of the expression levels of BOULE.

## Discussion

SG formation is one of early responses to heat stress in the male germ cells [20]. DAZL is a germ cell-specific component of SGs [27]. In this study, we show that BOULE, the founding member of DAZ family proteins, is also a SG component in mouse male germ cells. Together, these results suggest that both of DAZ family proteins in the mouse may share similar roles in SGs.

It is known that SGs store translationally inert mRNAs and associated RNA-binding proteins under stress conditions [21]. A number of mRNAs, including *Mvh* (*mouse vasa homologue*), *Sycp3* (*synaptonemal complex protein 3*) and *Tex19*.*1* (*testis expressed gene 19*.*1*), have been identified as mRNA targets of DAZL [32–34]. *Cdc25/twine* is known as a putative target of BOULE [35]. Interestingly, all of these genes are essential for meiotic progression and male germ cell development [35–38]. It is expected that a set of mRNAs bound to DAZL or BOULE is also recruited to SGs upon heat stress. These target mRNAs in SGs are likely to be temporarily inactive during heat stress conditions.

Even if BOULE participates in SGs upon heat stress, this localization to SGs is limited to the germ cells at specific developmental stages–early pachytene spermatocytes of stages III-VI. We observed that BOULE remained diffuse in the cytoplasm of diplotene and meiotic spermatocytes and early round spermatids in which SGs were not formed. By contrast, DAZL-expressing germ cells well overlap with the SG-forming cells [27]. These results suggest that, in contrary to DAZL which is required to form SGs, BOULE and its bound mRNAs may be passively recruited to SGs.

In male germ cells, SG formation appears to be developmental stage-specific and thus is likely dependent on the cell cycle. A study using cultured cells after UV irradiation has shown that SG formation is a cell-cycle dependent event [39]. So it is possible that only a set of germ cells in specific phases of the meiotic cell cycle has an ability to form SGs. However, why SGs are formed in a specific set of cells that seems to be tolerant during heat stress is still an open question.
